# Analyzing the Effects of UAV Mobility Patterns on Data Collection in Wireless Sensor Networks

**DOI:** 10.3390/s17020413

**Published:** 2017-02-20

**Authors:** Sarmad Rashed, Mujdat Soyturk

**Affiliations:** Department of Computer Engineering, Faculty of Engineering, Marmara University, 34722 Istanbul, Turkey; sarmadkadimrashedrashed@marun.edu.tr

**Keywords:** unmanned aerial vehicle (UAV), cluster head, mobility pattern, coverage problem, utilization, quality of service

## Abstract

Sensor nodes in a Wireless Sensor Network (WSN) can be dispersed over a remote sensing area (e.g., the regions that are hardly accessed by human beings). In such kinds of networks, data collection becomes one of the major issues. Getting connected to each sensor node and retrieving the information in time introduces new challenges. Mobile sink usage—especially Unmanned Aerial Vehicles (UAVs)—is the most convenient approach to covering the area and accessing each sensor node in such a large-scale WSN. However, the operation of the UAV depends on some parameters, such as endurance time, altitude, speed, radio type in use, and the path. In this paper, we explore various UAV mobility patterns that follow different paths to sweep the operation area in order to seek the best area coverage with the maximum number of covered nodes in the least amount of time needed by the mobile sink. We also introduce a new metric to formulate the tradeoff between maximizing the covered nodes and minimizing the operation time when choosing the appropriate mobility pattern. A realistic simulation environment is used in order to compare and evaluate the performance of the system. We present the performance results for the explored UAV mobility patterns. The results are very useful to present the tradeoff between maximizing the covered nodes and minimizing the operation time to choose the appropriate mobility pattern.

## 1. Introduction

Proliferating real-world implementations of wireless sensor networks (WSNs) and an abundant number of studies for decades have proven that WSNs are one key enabling technology for monitoring environments, not only in military, but also in industry and civil areas [1]. On the other hand, large or remote area deployments introduce additional challenges, such as connectivity and maintenance, in addition to the well-known issues such as poor communication environment, limited resources (battery, central processing unit (CPU), and memory), and limited bandwidth. To cope with these challenges, clustering is one promising solution applied in WSNs [2,3].

In large-scale and remote area deployments, data acquisition appears as another major challenge [4]. Long distances between the source and the sink nodes prevent direct communication. Although multi-hop communication is a promising solution achieved by routing the data to the sink node, it brings performance degradation and increases the cost in large-scale deployments. Instead, the use of a mobile sink node that moves within the network to collect data is a better solution with respect to the use of a stationary sink node [5,6,7,8]. Considering the speed of the mobile sink node and the size of the remotely monitored areas, Unmanned Aerial Vehicles (UAVs) are the best option to cover all area in a short period of time [9,10,11,12].

UAVs have some limitations; for example, operation altitude, speed, available radios, and carrying capability. The endurance time of the UAV can be limited, which may force the UAV to complete its mission within a certain amount of time. To provide a radio link connection with the sensor nodes, the UAV should be mounted with the same type (identical) of radio as the sensor nodes. Identical radio usage forces the UAV to fly at an altitude that is accessible by the sensor nodes. To gather the data from each node, the UAV has to cover the entire operation area without leaving any sensor node unvisited. The time to cover all nodes might be longer than the endurance time of the UAV; as a consequence, the UAV may leave out some uncovered nodes. Therefore, there is a need to explore the behavior and performance of the network in case of the use of various UAV mobility patterns. Our hypothesis is that, depending on the density of the network and the size of the operation area, a better UAV pattern might be determined.

UAV mobility patterns have major effects on the coverage and connectivity of WSN [11,13,14]. The coverage and connectivity can be considered to measure the Quality of Service (QoS) of the Wireless Sensor Network, and the accuracy of the information collected from the deployed nodes. Therefore, UAV mobility pattern affects the performance of the network. The main aim in the network is data acquisition from the deployed WSN. However, due the limitations mentioned, the UAV might not cover all nodes in the operation area. There could be some nodes which might not access the UAV to send its data. The UAV may follow a path laid out to reduce the number of uncovered nodes. The UAV flight pattern should also consider the UAV endurance time. Therefore, there is a tradeoff between the operation time of the UAV and the covered nodes. If the effects of the mobility pattern of the UAV on the performance of the network are analyzed, a better mobility pattern can be found.

On the other hand, clustering in WSN allows the cluster heads (CHs) to send the aggregated cluster data to the UAV. Therefore, even a sensor node may not have access to the UAV, but to a CH that has UAV access, will have indirect access to the UAV. It is clear that clustering in WSN underneath the UAV path will improve the connectivity in the WSN and enhance the data acquisition [12].

Our aim in this paper is to explore the effects of the UAV mobility patterns on the acquisition of the data from the WSN. We modeled various mobility patterns and observed the effects of the mobility pattern on data collection. The contribution of this paper can be summarized as follows:

- Exploring UAV mobility patterns for covering the sensor nodes in the application area.
- Analyzing the effects of clustering and the UAV mobility patterns on clustering (e.g., the number of clusters formed).
- Exploring the mobility patterns for the minimization of the UAV operation time and for the maximization of the number of covered nodes.
- Providing insights about the mobility patterns and their effects on the clustering algorithm.
- Increasing the perception of the UAV operator for the decision of the appropriate UAV operation parameters under given constraints (e.g., time).

This paper is arranged as follows. In the second section, motivations of this work and the related work are presented. The network model and the mobility patterns are defined in the third section. Performance results and the simulation environment are given in the fourth section. The fifth section is reserved for the conclusions.

## 2. Motivation and Related Work

### 2.1. Motivation

The use of a mobile sink is a better solution for the collection of data in large-scale WSNs, especially remotely-controlled areas [5,6,8,11,15,16]. The mobile sink follows a path to provide load balancing and connectivity, in addition to the aim of accessing each sensor node. On the other hand, coverage and connectivity problems may persist due to the limiting factors (e.g., sink node speed, operation area size) affecting successful data acquisition from the remote area. Forming clusters is an appropriate solution to reduce the communication needs between sensor–sink pairs while enhancing the connectivity and coverage. There are clustering algorithms in the literature that aim to enhance coverage and connectivity [17,18], but the results show that a number of nodes still remain uncovered while some nodes declare themselves CH without any member nodes. These CHs with no member nodes are named as “standalone nodes”. Standalone nodes degrade performance by affecting the balance among the clusters and the network.

Recent studies have revealed that the use of UAVs is an effective solution for the purpose of monitoring and data collection from remote areas in a short period of time [11,19]. Moreover, in conjunction with clustering algorithms which consider the UAV flight path, more stable clusters can be formed within the WSN. In this kind of UAV-integrated WSN, two major limiting factors appear that affect the UAV operations: (1) UAV endurance; and (2) the requirement of identical radio usage with sensor nodes. Identical radio use limits the operation altitude, while the UAV endurance limits the operation time of the UAV. In large-scale WSN, the time required to cover all nodes might be longer than the endurance time of the UAV, which may leave some nodes inaccessible [20]. The UAV mobility pattern plays a key role in reducing the number of inaccessible nodes and in using the endurance time more efficiently. There is a need to explore the effects of various UAV mobility patterns in the formation of clusters underneath and in the data collection from the WSN.

### 2.2. Related Work

Several algorithms have been proposed to cover the maximum number of nodes by forming clusters; they are known as clustering algorithms. In these approaches, the main aim is forming well-balanced clusters and avoiding standalone nodes. In clustering algorithms, clusters are formed based on the cluster head (CH), so there is a cluster head election algorithm which affects the overall performance. Other objectives of the clustering algorithms are enhancing end-to-end delay, fault tolerance, balancing the load within the network, and increasing the connectivity. One of the early studies in clustering is Low-Energy Adaptive Clustering Hierarchy (LEACH) [17,21]. In LEACH, a distributed clustering algorithm is used to form the clusters. Cluster Head nodes are selected randomly with a probability value. This algorithm does not guarantee the formation of stable clusters, or the same number of clusters at each round. Hybrid, Energy-Efficient, Distributed clustering (HEED) [18,22] is another distributed clustering algorithm. HEED aims to form more balanced clusters while reducing the energy consumption by considering several parameters. Cluster head election uses residual energy at nodes to select the best node as CH. Extended HEED [23] is an extension to the HEED. It uses a core extraction algorithm to reduce the number of standalone CHs, which is very high in number in HEED. Received Signal Strength Indicator (RSSI)-based Hybrid and Energy-Efficient Distributed clustering (rHEED) [12] is a multi-hop distributed clustering algorithm and improves upon HEED [18]. It uses the Received Signal Strength Indicator for cluster formation and cluster head selection. It has been tested in an environment where the UAV takes the role of mobile sink. It is seen that more nodes are covered with the use of rHEED compared to the others, so in this study, this algorithm has been used to form clusters in WSN.

Recently, there have been many studies on data collection from WSNs with the use of UAVs. In [24], authors present a cross-layer transmission approach—namely, Adaptive-OAloha—for efficient communication between sensor nodes and UAVs. A dynamic priority assignment-based Slotted Aloha scheme is used for the communication. The UAV followed the predefined (tractor) trajectory on data collection. The main aim of the research is reducing the energy consumption at nodes.

In [25], authors also aimed to gather the sensing data from WSN with the use of a UAV. Authors proposed a cooperative data collection scheme for a WSN, in case each sensor node may not able to convey its data to the centralized storage (e.g., cloud), but utilizes the UAV for cooperative data transfer. Therefore, Medium Access Control (MAC) protocol is proposed for making up the decision criteria of the cooperative communications between the cooperative sensor nodes. The probability of correctly receiving sensing data is calculated through Monte Carlo simulations in which a UAV follows a tractor mobility pattern on the operation area.

In [26], authors proposed an algorithm for path planning of aerial vehicles to collect the data from WSNs. After sensor network deployment, nodes learn their position either by localization or Global Positioning System (GPS). Positions of the sensor nodes are obtained at the data center to determine the anchor nodes. A flight path is calculated considering the position of anchors, and the UAV only visits the selected sensor nodes (anchor nodes). The proposed path planning algorithm is a solution similar to the Traveling Salesman Problem. In the simulations, authors presented results on sensor node coverage. Authors also concluded that the anchor node selection has major impact on UAV path and the network performance. They also signified the dependency of their approach on the GPS for positioning and anchor node selection.

A very similar approach is presented in [27], where Particle Swarm Optimization (PSO) is used to find an optimized UAV flight path for visiting selected sensor nodes. Different than [26], the proposed approach forms clusters and the UAV visits the elected cluster heads. Similar to the previous approach, the proposed clustering method and path planning algorithm are dependent on the node positions, and the UAV must know the location of the CHs before the mission starts. In the evaluations, the proposed approach is compared with LEACH-centralized (LEACH-C) through Monte Carlo simulations.

As the UAV-based solutions are attractive for both military and industry, a need to prevent possible collisions in the air appears. Authors in [28] presented a real-time flight monitoring and management scheme considering the UAV paths, positions, and other sensory measurements. The proposed solution prevents potential collisions and recommends adjustments by alerting the users.

For a team of UAVs, authors in [29] aim to determine the positions of UAVs over the WSN and aim to assign sensor nodes to the UAVs. The main goal is to solve the connectivity problem between the UAVs and between the UAV and sensor nodes with the use of integer programming algorithms.

Another path optimizing study is proposed in [30]. The main aim is to control the amount of chemicals sprayed by the UAV over the agricultural area. Due to the wind and other conditions, the amount of chemicals on the crop might vary. Therefore, a sensor network is formed in the operation area to inform the UAV immediately about the amount of perceived chemicals. In response, the UAV immediately adjusts its route to spray the required amount of chemicals on the crops.

An implementation of UAV for data gathering from the WSN located in agricultural areas is presented in [31]. Authors deployed a WSN in an agricultural area and instrumented an UAV for the purpose of collecting data from the WSN, streaming live video and images from the area, detecting disease or pests in crops, and spraying substances or chemicals on the infected crops or pests.

In [32], for the purpose of post-disaster monitoring, authors presented design strategies using UAVs to deploy a WSN in the area. The main focus was on navigation of the UAV and localization of the deployed sensor nodes. Complementarily, authors in [33] overviewed the possible disaster management applications of UAV networks and discussed open research issues related to the use of UAVs.

In [34], a data collection method is proposed that makes use of mobile agents. A UAV dispatches the mobile agents while flying over the WSN. Mobile agents move from one sensor node to another, perform the tasks requested by the UAV, and collect the data at nodes. In the next run, mobile agents return back to the UAV. The UAV has to follow a path to collect mobile agents from the operation area.

An interesting work has been presented in [35] which presents the models and the strategies for efficient data collection from the WSN by the UAV. Selected relay nodes among clustered sensor nodes transmit the collected data to the UAV. The authors present various strategies to enhance data collection among sensor nodes. These strategies include the UAV moving with constant versus variable versus adaptable speed, hovering with limited versus maximum service time, and collecting the data continuously in round-robin fashion or on-demand.

In the presented UAV-integrated solutions, the UAV followed either the predefined path/mobility pattern or the UAV path was determined based on the predefined visiting points. As far as we know, there is no work in the literature that observes the effects of UAV mobility patterns on data collection in WSNs. Furthermore, there is no work where the UAV mobility pattern and the clustering algorithm are merged to optimize the data collection and other performance metrics.

## 3. Network Model and Explored Mobility Patterns

**Figure 1:**
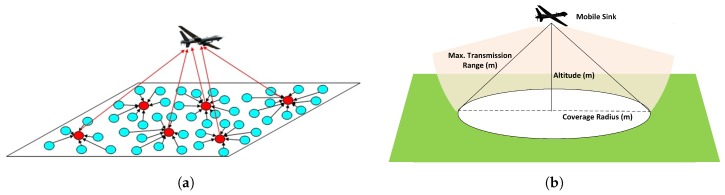
(**a**) Network Model; (**b**) Instant sweep (coverage) region of the unmanned aerial vehicle (UAV) [12].

In the network model, we assume that the nodes are randomly distributed in the remotely accessible operation area, as shown in Figure 1a. Sensor nodes in the WSN apply a clustering algorithm where a CH is elected in each cluster in the network. A single UAV operates in the operation area to collect data from the WSN. Only the CHs interact with the UAV to reduce the overhead and to provide energy efficiency. We also assume that clusters are formed considering the path of the UAV, which is known or learned by the nodes. The rHEED [12] algorithm is an example of this kind of clustering algorithm.

**Figure 2:**
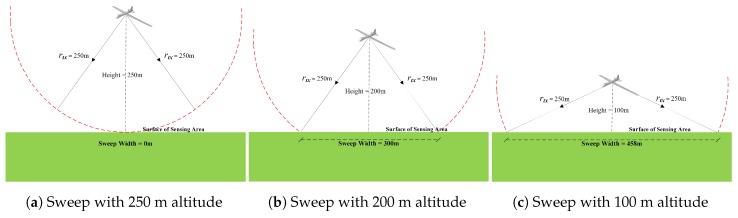
Illustration of the sweep width with different UAV altitudes. Sweep width depends on the altitude of the UAV.

While the UAV moves over the operation area, an instant sweep region is constructed on the ground, as depicted in Figure 1b. The instant sweep (coverage) region varies depending on the altitude of the UAV and the transmission range (or transmission power of the UAV and sensor nodes). As shown in Figure 2, as the UAV altitude decreases, the instant sweep region enlarges, which allows more nodes to be covered at low altitudes. Therefore, the altitude and transmission power are important parameters for accessing more nodes in the area. For the purpose of improving the connectivity in the WSN, we assume that there is a clustering algorithm (e.g., rHEED) applied at nodes considering the UAV path. Clusters are formed over/near the UAV path, while only the CHs are allowed to make direct access/communication to the UAV. Member nodes in clusters will communicate with their CHs. As result, even though a cluster member node may not have direct access to the UAV, it will be covered indirectly if its CH has access to the UAV. Nodes in the area form clusters and elect CHs based on the mobility pattern of the UAV.

Well-known and practically implemented mobility patterns are discussed below, which will be used further in the evaluations.

### 3.1. Tractor Mobility Pattern

**Figure 3:**
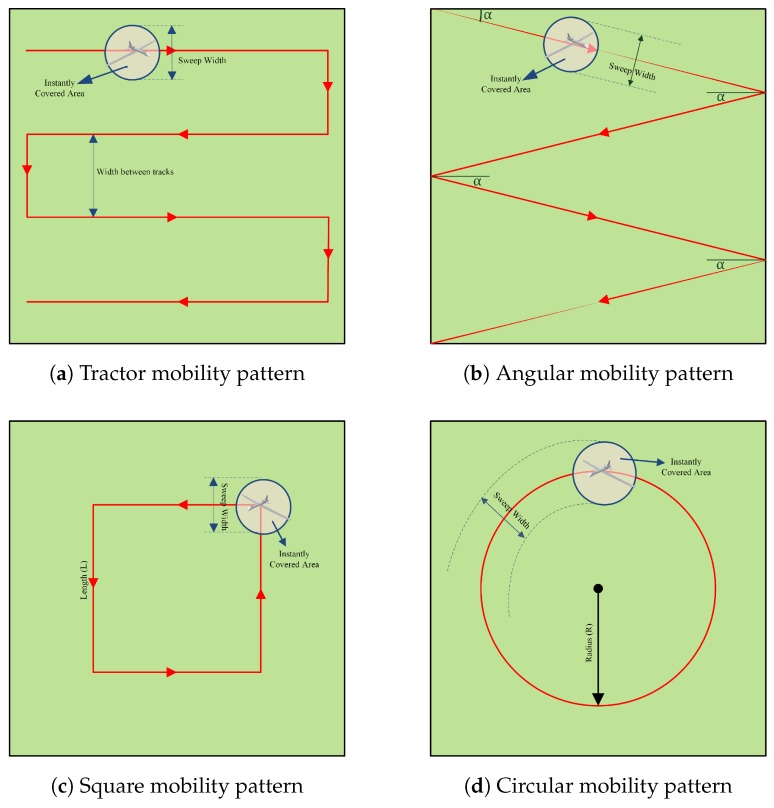
Illustration of explored mobility patterns.

In this mobility model (as shown in Figure 3a), the UAV follows parallel tracks. As the width between the tracks gets wider, the number of tracks decreases. To cover all nodes without leaving any gap, the width between tracks should be kept at minimum, while the wider widths may leave gaps between the tracks. When the UAV arrives to the operation area, it moves to the starting point and follows the tracks. Narrower widths increase the number of tracks, and so the path length and the time to complete the mission. On the other hand, wider width between them may lead to some uncovered nodes.

### 3.2. Angular Mobility Pattern

In this mobility model (as shown in Figure 3b), the UAV follows angular tracks. On arriving to the border of the operation area, the UAV turns its direction to the reverse with a reflection from the border, with an angle value. When the UAV arrives to the operation area, it moves to the starting point and follows the angular path. Small angle values increase the number of legs, and so the path length and the time to complete the mission. On the other hand, large angles may leave gaps and may cause some uncovered nodes.

### 3.3. Square Mobility Pattern

In this mobility model (as shown in Figure 3c), the UAV follows a rectangular path. The center of the rectangle/square is the center of the operation area. As the side length of the rectangle gets smaller, smaller rectangles are drawn. Large rectangles leave a gap at the center of the rectangle, and small rectangles leave gaps outside of the rectangle. When the UAV arrives to the operation area, it moves to the starting point and follows the rectangular path. Larger rectangles increase the circumference, and so the side length and the time to complete the mission. On the other hand, small rectangles may leave gaps and may cause some uncovered nodes.

### 3.4. Circular Mobility Pattern

In this mobility model (as shown in Figure 3d), the UAV follows a circular path with an angle to draw a circle. The center of the circle is the center of the operation area. As the angle of the curve gets smaller, smaller circles are drawn. Large circles leave a gap at the center of the circle, and small circles leave gaps at the corners of the operation area. When the UAV arrives to the operation area, it moves to the starting point and follows the circular path by turning to one side with the same angle value. Larger circles increase the circumference, and so the path length and the time to complete the mission. On the other hand, small circles may leave gaps and may cause some uncovered nodes.

## 4. Simulation and Results

### 4.1. Simulation Environment

We modeled and simulated the network with OMNET++ [36] and MiXiM [37,38]. Wireless sensor nodes were equipped with IEEE 802.15.4 compliant TI CC2420 radio [39]. The radio is realistically modeled in MiXiM with TI CC2420 NIC which contains a physical (PHY) layer and a medium access control (MAC) layer to interact with the network (NETW) layer. The UAV has also been equipped with the same type of radio (TI CC2420) in order to be able to communicate with the CHs. Among the UAV models, the characteristics of Bayraktar [40] have been modeled and implemented. This type of UAV has the ability to operate at low altitudes with long endurance duration. Nodes in the network dispersed randomly over a 2000 m × 2000 m area. The UAV mounted with on-board GPS flies over the operation area and sweeps the area with the modeled mobility patterns, which are defined in Section 3. In the WSN, nodes form clusters with the rHEED [12] algorithm. Only the CHs and/or standalone nodes communicate with the UAV, while the cluster members send their data to their own CH. Clusters are formed depending on the path of the UAV. Because the nodes cannot know the path of the UAV ahead of time, with a realistic scenario, the UAV makes a blind run over the operation area. Nodes in the WSN record the RSSI value of the UAV beacons to use in CH election and cluster formation. Clusters are formed after the blind run, and then in the actual run, CHs send their data to the UAV as defined in [12]. The UAV flies with a constant speed of 20 m/s, maximum altitude of 250 m, path loss exponent 2.5. The nodes’ receiver sensitivity is set to −95 dBm. Table 1 shows the simulation parameters.

### 4.2. Performance Metrics

For a node to be able to send its data to the UAV, it must either have a direct link with the UAV, or it must be a member of a cluster where the CH has direct access to the UAV. Therefore, for data collection in the WSN, coverage of the operation area is directly related with the data collection. If a node does not have a direct or indirect path to the UAV, then it will not be able to forward its data to the UAV. Hence, the data collection problem turns out to be a coverage problem.

The UAV mobility patterns have major effects on the coverage and connectivity in the operation area. The coverage and connectivity can be considered to measure the QoS of the WSN and the accuracy of the information collected from the deployed nodes. On the other hand, the time elapsed to collect the data from the WSN is another important metric and aspect. The aim should be to collect as much data as possible in the shortest possible period of time. Therefore, in our experiments, the following six metrics are analyzed to see the effects of the UAV mobility pattern on the coverage of the operation area:

- *The Number of Covered Nodes* is the total number of covered nodes in the operation area. It is calculated as the sum of (1) CHs which have access to the UAV, (2) cluster member nodes which have access to a CH that has access to the UAV, and (3) standalone nodes which have no access to any CHs, but have direct access to the UAV.
- *The Number of CHs* is the total number of CHs with or without member nodes (standalone CHs). It gives the number of formed clusters.
- *Time Spent to Cover a Single Node* is the time the UAV spent in the operation area divided by the covered nodes.
- *Utilization* is the ratio of the covered nodes to the total nodes deployed in the operation area. It gives the coverage efficiency.
- *Time and Coverage Efficiency* is the ratio of the covered nodes to the time elapsed to cover those nodes.
- *Normalized Time and Coverage Efficiency* is the normalized value of the Time and Coverage Efficiency. Each pattern may perform differently in terms of covered nodes and time efficiency. In order to use a general metric and scale, the normalized value is much more appropriate.

Each mobility pattern has been tested with various values of the mobility pattern characteristics (e.g., various radius values for circular mobility). Each mobility pattern has also been tested with various altitude values (100 m, 150 m, 200 m, and 250 m). UAV altitude effects the connectivity between the nodes in the application area and the UAV, where connectivity consequently has an effect on other performance metrics, as described in next section. All results are the averages of at least 10 runs to confirm 95% confidence level.

### 4.3. Performance Results

In making decision to select the best appropriate mobility pattern to apply in the operation area, there could be two main objectives: (1) covering the maximum number of nodes; (2) spending less time in the operation area with maximum utilization. Therefore, the performance results are based on the measurements to observe the effects of mobility patterns on these objectives.

#### 4.3.1. Coverage

The number of cluster heads represents the number of clusters formed. Although a smaller number of clusters is admired in WSN, in such large-scale operation areas where the coverage is the major problem, fewer clusters may indicate a high number of uncovered nodes. Therefore, although the number of cluster heads gives some valuable information, it has to be evaluated together with the number of covered nodes.

**Figure 4:**
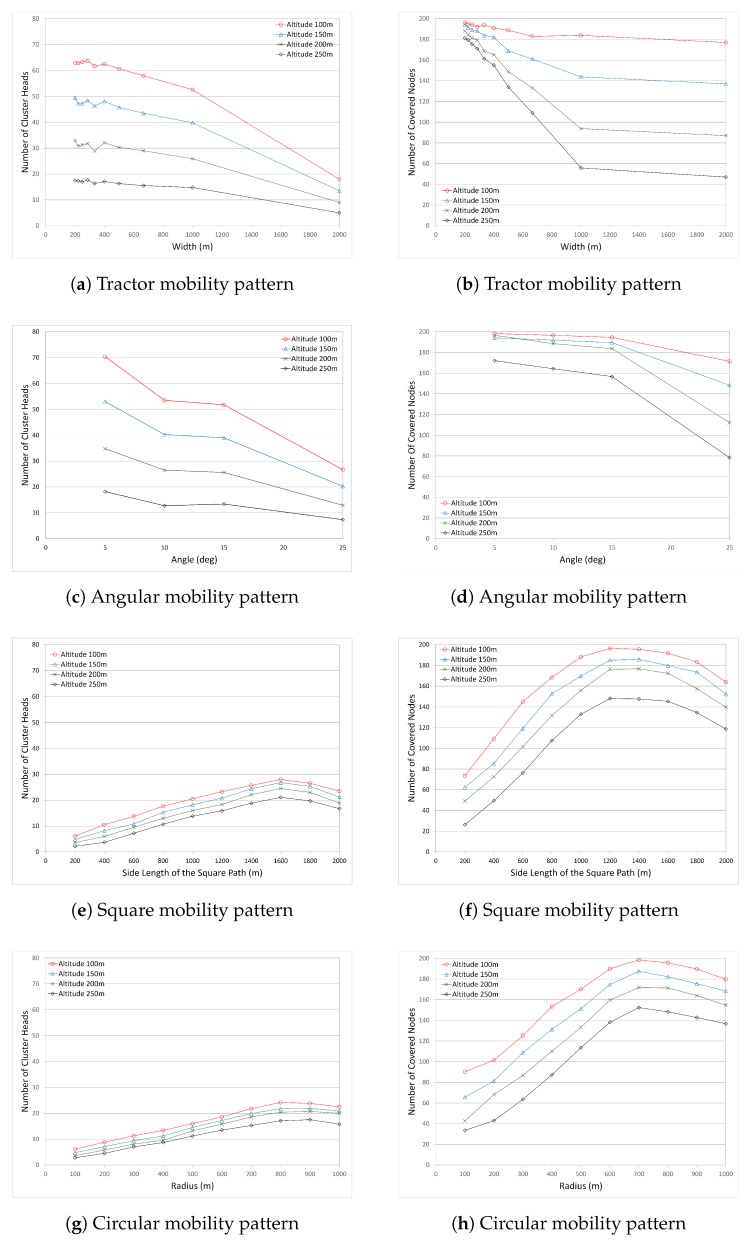
Comparison of the number of cluster heads (CHs) and the number of covered nodes for all mobility patterns.

The coverage in the Tractor mobility pattern depends on the width between the parallel tracks. Coverage with track mobility is tested with various track widths between 200 m and 2000 m. With 2000 m width, the UAV crosses the operation area at the center from one end to the other. As the track width narrows, the number of tracks increases, which leads to an increase in the UAV path length. Longer paths also lead to the coverage of more sensor nodes and the formation of more clusters closer to the UAV path. On the other hand, with fewer tracks, gaps are expected to exist with some uncovered nodes between tracks. As expected, Figure 4a shows that increasing the width of the tracks leads to a decrease in the number of CHs. The maximum number of CHs with UAV access is seen at width value 200 m with the UAV altitude value 100 m. For the 250 m altitude, the number of clusters are at a minimum, and only the sensor nodes under the exact projection of the UAV path can be the CHs. Although the number of CHs is a good indicator of coverage, more accurate results are obtained by the number of covered nodes. Figure 4b shows the number of covered nodes in the Tractor mobility pattern. It is seen that when the track width is narrow, the number of covered nodes are very close to each other for each altitude. However, when the track width increases to more than 400 m, the number of covered nodes decreases sharply (except with 100 m altitude), with fewer covered nodes for higher altitudes. When the two figures are compared, it is seen that the number of covered nodes are not directly proportional to the number of CHs. Although the number of CHs for altitude 250 m is steadily low for all width values, the number of covered nodes does not present similar results. The reason is that selected CHs form clusters with their member nodes, which are considered to have indirect access to the UAV through the CHs. Therefore, altitude 250 m performs results closer to other altitudes when the track width is narrow. The clustering algorithm plays a key role in the formation of clusters based on the UAV path. This leads to more nodes being covered, even though they might not have direct access to the UAV. This characteristic is also seen at altitude 100 m. Although the number of CHs decreases sharply when the width is about 500 m, the number of covered nodes does not decrease sharply, almost having steady and similar results. It is also seen in Figure 4b that for altitudes other than 100 m, as the width grows to 1000 m, the number of covered nodes decreases sharply, but after 1000 m it starts to decrease slightly. The reason is related to the gaps between the tracks. When the width is small, there is no gap between the tracks where the UAV covers most of the nodes. As the width grows, gaps get larger, causing the decrease in the number of covered nodes.

In the Angular mobility pattern, the UAV is reflected from the border with the defined angle value when the UAV impinges on the border of the operation area. Therefore, the coverage in the Angular mobility pattern depends on the angle value. The mobility pattern is tested with four different angle values: 5, 10, 15, and 25. It is seen in Figure 4c that increasing the angle value leads to a decrease in the number of CHs. With smaller angles, the number of legs on the path is increased; therefore, more clusters are formed closer to the UAV path. On the other hand, it is expected to see some gaps between the legs when the angle gets larger (Figure 3b). The maximum number of CHs with UAV access is seen at an angle value of 5${}^{\circ }$. On the other hand, decreasing the altitude also leads to an increase in the number of CHs. This is related to the coverage and sweep width, as illustrated in Figure 2. At low altitudes, more nodes receive the signals from the UAV to compete to be CHs. Figure 4d shows the number of covered nodes in the Angular mobility pattern. It is seen that the number of covered nodes decreases as the angle value increases. Smaller angle values cause fewer UAV legs to be constructed over the operation are, which leads the UAV to sweep less of an area. At the angle value 5${}^{\circ }$ and altitude 100 m, 197 out of 200 nodes are covered with the maximum value. For all angle values except 25${}^{\circ }$, the number of covered nodes are very close to each other. However, at the angle value 25${}^{\circ }$, the number of covered nodes reduced significantly—especially for the altitudes 250 m and 200 m. When the covered nodes are compared according to the altitude, it is clearly seen that low altitude covers more nodes. Very similar to the Tractor mobility pattern results, the number of covered nodes are not directly proportional to the number of CHs. There is a sharp decrease in the number of CHs between angles 5${}^{\circ }$ and 10${}^{\circ }$, but the number of clustered nodes does not change much. The reason is related to the clustering algorithm. Although the number of clusters is reduced with higher angle values, clustering algorithms are able to connect member nodes to the UAV with the use of CHs. Therefore, the number of covered nodes is not affected much with respect to the CHs when the angle value is small. On the contrary, when the angle value is very large (e.g., 25), the number of covered nodes reduces due to the gaps between legs.

The coverage in the Square mobility pattern depends on the length of the edge of the square, where the center of the square is the center of the operation area. The mobility pattern was tested with 10 different square side lengths, starting from 200 m to 2000 m, with 200 m increase at each step. With 2000 m side length, the UAV follows the borders (edges) of the square-shaped operation area. It is important to see the effects of gaps in the center when the square is large and the effects of gaps at the outside of the square-shaped sweep region when the square is small. With a wider square, the length of the path is increased; therefore, more clusters are formed closer to the UAV path. The maximum number of CHs with UAV access is seen at side length value 1600 m at all altitude values, and the maximum is reached with altitude 100 m (Figure 4e).

Figure 4f shows the number of covered nodes in the Square mobility pattern. At side length 1200 m and altitude 100 m, most of the 200 nodes are covered. It is seen that as the square path increases, the number of covered nodes increases until the side length value 1200 m, and then decreases slightly. The reason is related to the gaps inside and outside of the square. When the side lengths (and therefore the squares) are small, there are gaps outside of the square where the UAV cannot cover any node in that region. As the size of the square grows, gaps outside of the square get smaller. On the other hand, after 1200 m, the gaps outside of the square are at a minimum or no gap exists, but new gaps appear at the center of the square. This is why the number of covered nodes increases until side length value 1200 m and decreases after that. When the altitudes are compared with each other, it is seen that all altitudes perform similarly, with some constant difference between each other. It is also noticeable that the performance of the Square mobility pattern is very similar to the Circular mobility pattern.

The coverage in the Circle mobility pattern depends on the radius value ***R***, where the center of the circle is the center of the operation area. It is seen in Figure 4g that increasing the radius of the circling UAV leads to an increase in the number of CHs. With a larger circle, the length of the path is increased; therefore, more clusters are formed closer to the UAV path. On the other hand, it is expected to see some gaps at the corners of the area when the circle gets smaller, and gaps at the center of the area when the circle gets larger. These gaps increase the number of standalone CHs (CHs without a cluster member) and uncovered nodes. The maximum number of CHs is seen at radius values 800 m and 900 m. As the altitude decreases, the UAV sweeps a greater area; therefore, the highest number of clusters is observed at 100 m with 800 m circling radius. Figure 4h shows the number of covered nodes. It is seen that as the circular path grows, the number of covered nodes increases until the radius value 700 m, and then decreases slightly. The number of covered nodes is proportional to the number of CHs. Similar to the Square mobility pattern, when the altitudes are compared with each other, it is seen that all altitudes perform similarly with some constant difference between each other.

When all mobility patterns are compared, the maximum number of covered nodes is seen at the altitude of 100 m. In the Angular mobility pattern, the number of covered nodes is not affected as much as other mobility patterns due to the varying performance parameters, altitude, circle radius, etc.

#### 4.3.2. Time Efficiency and Utilization

On data collection in the WSN, one main aim is to collect the data from as many nodes as possible, and the other aim is to collect the data as quickly as possible to reduce the operation time. In large-scale WSN deployments, every node in the network might not be accessed without leaving a gap in the network. Accessing each sensor and retrieving its data is usually the main aim. However, this approach might introduce additional UAV operation time to be spent in the operation area. Covering each sensor node may cause unnecessary cost, which might even not be fulfilled by the UAV capabilities. Therefore, it is essential to observe the time efficiency to gather the required information from the WSN. It is essential to know how much more time is required to cover some more nodes. If the time required to cover a few more nodes will increase the overall time too much (in other words, if the benefit remains marginal), the intention of covering some more nodes might be prevented to keep the cost at an acceptable level and to utilize the time and cost more efficiently. For the time efficiency, the Time Spent per Covered Node metric is defined, and is formulated as follows:

(1) $$\mathrm{Time}\;\mathrm{Spent}\;\mathrm{per}\;\mathrm{Covered}\;\mathrm{Node}=\frac{\mathrm{the}\;\mathrm{time}\;\mathrm{UAV}\;\mathrm{spent}\;\mathrm{in}\;\mathrm{the}\;\mathrm{operation}\;\mathrm{area}}{\mathrm{the}\;\mathrm{number}\;\mathrm{of}\;\mathrm{covered}\;\mathrm{nodes}}$$

**Figure 5:**
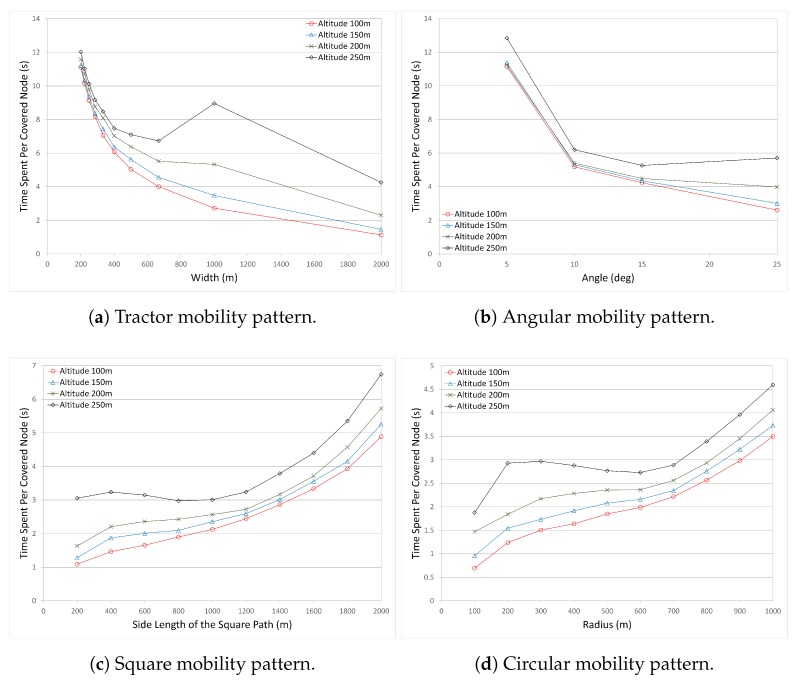
Comparison of the Time Spent Per Covered Node for all mobility patterns.

Figure 5 shows the time spent per covered node for all mobility models with various altitudes. Figure 5a shows the time spent to cover a single node in the Tractor mobility pattern. In the Tractor mobility pattern, as the path length decreases (the width between the tracks increases), the time spent to cover a single node decreases sharply until width value 500 m, between 500 m–1000 m, there is a moderate decrease, and after 1000 m, the time spent per covered node starts to decrease slightly. The results for altitude 250 m differ from the other altitudes. At altitude 250 m, the time spent per covered node decreases sharply, then starts to increase again at 700 m, and starts to decrease after 1000 m. It is related with the number of covered nodes. According to these results, the time spent per covered node is very small at the width value 2000 m, but it is most probably an inadvisable parameter to use when the number of covered nodes is also considered.

Figure 5b shows the time spent to cover a single node in the Angular mobility pattern. It is seen that as the angle value increases (path length decreases), the time spent to cover a single node decreases sharply, and then continues to decrease slightly after angle value 10${}^{\circ }$ for all altitude values. At angle value 25${}^{\circ }$ and altitude 100 m, the time spent to cover a single node is at a minimum. Except altitude 250 m, all altitudes present similar results. Results for 250 m are much higher compared to the others. The reason is related to the number of covered nodes. On the other hand, it is seen that the minimum time spent per covered nodes is observed at angle value 25${}^{\circ }$ at all altitudes. However, when we compared with the number of covered nodes, angle value 25${}^{\circ }$ performs the worst in terms of the number of covered nodes. In this case, there is a dilemma in choosing the best mobility model and pattern; that is, whether the number of covered nodes or the time spent per covered node should be selected as the decision metric. Therefore, a new metric Time and Coverage Efficiency is defined, and will be presented later.

Figure 5c shows the time spent to cover a single node in the Square mobility pattern. It can be seen that when the side length (and therefore the path length) increases, the time spent to cover a single node increases slightly until length value 1200 m, and then starts to increase sharply. The results for the Square mobility pattern are very similar to the results of the Circular mobility pattern, for which the results will be discussed in further detail.

Figure 5d shows the time spent to cover a single node in the Circular mobility pattern. When the UAV follows a path (i.e., circle), the path length depending on the mobility type becomes important. As the path length increases, the UAV will spend more time in the air. However, depending on the clustering algorithms and the UAV path, more stable and balanced clusters can be formed. Therefore, a preferred pattern can be selected based on the time per node. Here, as is seen in Figure 5d, when he circular path length increases, the time spent to cover a single node increases, then the increase rate slows down due to more covered nodes, and then increases again. The minimum time spent per covered node is observed at the radius 100 m. However, the same decision issue arises again. With the radius 100 m, the Circular mobility pattern performs the worst with respect to the number of covered nodes. Therefore, as mentioned in angular mobility, the number of covered nodes and the time spent to cover these nodes have to be considered together. According to these results, at radius between 500 m and 700 m, more nodes are covered within a shorter time. If we consider only the number of covered nodes, the altitude 700 m is the best choice in circular mobility.

Among all mobility patterns, the Square mobility pattern and the Circular mobility pattern have lower and more stable values for the time spent per covered node. It is also seen that the rate of change in the Square and Circular mobility patterns is lower than the Tractor and Angular mobility patterns.

When all mobility patterns are compared, the time spent per covered node in Angular and Tractor mobility patterns is very high for some values of angle and track width (i.e., angle value 5${}^{\circ }$ in Angular mobility pattern and track width value 200 m in Tractor mobility pattern). On the other hand, depending on the parameter values, the time spent per covered node reduces to very small values (i.e., for angle value 25${}^{\circ }$ in Angular and track width value 2000 m in Tractor mobility patterns). For these small values, it is questionable to use these parameters in the data collection of such a large-scale WSN. When we look at the number of covered nodes for the scenarios with these small values of time spent per covered node, it is seen that very few nodes are covered. So, it is the network operator’s responsibility to consider multiple metrics depending on the objectives. There is a tradeoff whether to use the parameters with more covered nodes or the parameters with short time to cover a single node. Altitudes affect the number of covered nodes at each mobility pattern, and the time spent per covered node is directly related to the number of covered nodes. This tradeoff introduces the utilization metric, which is considered as a measure for the performance of the system.

In the literature, there are many performance metrics defined to measure the performance of the system. Three of them are very close to each other. These are the utilization, the efficiency, and the productivity metrics. Their corresponding formulas are given below.

(2) $$\mathrm{Utilization}=\frac{\mathrm{the}\;\mathrm{number}\;\mathrm{of}\;\mathrm{covered}\;\mathrm{nodes}}{\mathrm{the}\;\mathrm{number}\;\mathrm{of}\;\mathrm{all}\;\mathrm{nodes}}$$

(3) $$\mathrm{Efficiency}=\frac{\mathrm{the}\;\mathrm{number}\;\mathrm{of}\;\mathrm{covered}\;\mathrm{nodes}\;(\mathrm{in}\;\mathrm{that}\;\mathrm{scenario})}{\max.\;\mathrm{number}\;\mathrm{of}\;\mathrm{covered}\;\mathrm{nodes}\;(\mathrm{in}\;\mathrm{all}\;\mathrm{scenarios})}$$

(4) $$\mathrm{Productivity}=\frac{\mathrm{the}\;\mathrm{number}\;\mathrm{of}\;\mathrm{covered}\;\mathrm{nodes}}{\mathrm{the}\;\mathrm{time}\;\mathrm{UAV}\;\mathrm{spent}\;\mathrm{in}\;\mathrm{the}\;\mathrm{operation}\;\mathrm{area}}$$

Productivity (4) is the inverse of the performance metric “The Time Spent per Covered Node” (1). Therefore, the results for the productivity will not be given because we have already given the results for the time spent per covered node.

**Figure 6:**
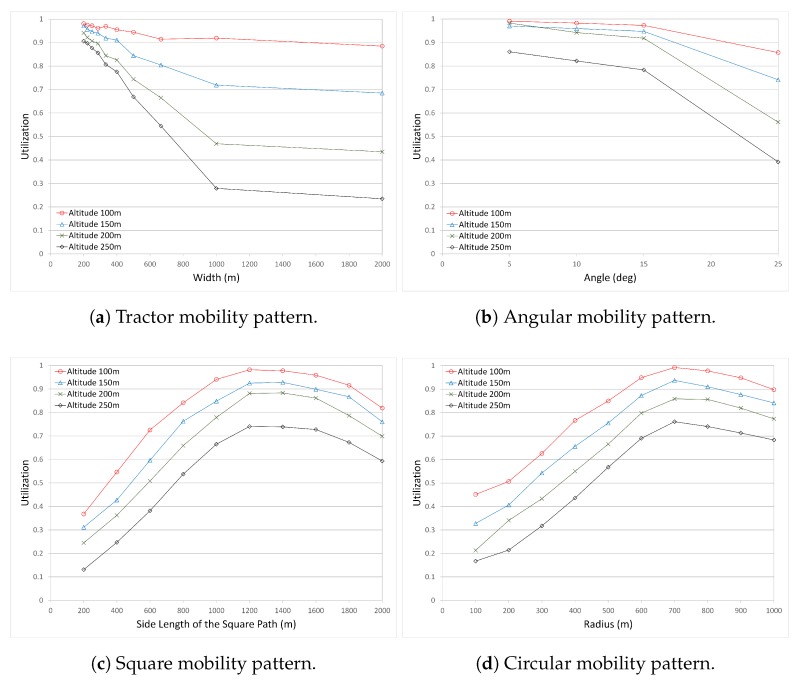
Comparison of the Utilization for all mobility patterns.

The Utilization (2) and the Efficiency (3) are very close to each other, giving similar results and the same curve pattern in the graphs. Their y-scales range between 0 and 1. Therefore, rather than using both metrics, we have used only utilization in the evaluations. Previously, the results for the performance metric “the number of covered nodes” were presented. It is clear that the utilization metric is the normalization of “the number of covered nodes”, where the utilization value varies between 0 and 1. Figure 6 presents the utilization results for all mobility patterns. The graphs for “the number of covered nodes” can be referred to when in need of more detailed information on each graph.

The utilization graphs of Circular and Square mobility patterns are very similar to each other (6). When the circle or square size is small, the utilization is also low. As the size of the circle and the square grows, the utilization increases. However, in both mobility patterns, the utilization starts to decrease after a peak value. This is because gaps begin to grow in the center of the circle/square.

In the Angular mobility pattern, the utilization is very high when the angle value is small for all altitude values. For the altitude 100 m, the utilization decreases slightly, but is very high compared to the other altitudes. In other altitudes, the utilization decreases slightly until angle value 15${}^{\circ }$, and decreases more at angle value 25${}^{\circ }$. Altitude 250 m has the worst utilization results. It is seen in Figure 6b that the altitude and the angle value affect the utilization. Small angle value and lower altitudes increase the connectivity and accessibility to the UAV.

The Tractor mobility pattern (Figure 6a) performs similarly to the Angular mobility pattern. For the UAV altitude 100 m, it performs better than other altitudes, and the utilization decreases slightly even as the width between tracks increases. However, other altitudes are affected strongly by the increase in the width of the tracks, and the utilization decreases sharply as the width and altitude increase.

#### 4.3.3. Time vs. Coverage Efficiency

In the metrics presented above, it is seen that considering only the coverage of the nodes or the time elapsed to cover the nodes is not enough, and may lead to false decisions being made. While maximizing the number of nodes is an important metric, minimizing the elapsed time is another important metric. The UAV flying over the operation area may not have enough energy to complete its mission. Or, the UAV may have limited time to collect as much of the necessary data as possible. Therefore, time and coverage metrics have to be analyzed together.

We define a new metric, which is a measure of the time and the coverage. It is named as “Time–Coverage Efficiency”. The aim is to maximize the number of covered nodes while minimizing the operation time. In other words, time spent for a node has to be minimized while maximizing the number of covered nodes. The equation for this new metric is given below:

(5) $$\begin{array}{c} \mathrm{Time}-\mathrm{Coverage}\;\mathrm{Efficiency}\approx \mathrm{maximize}\left\{\mathrm{the}\;\mathrm{number}\;\mathrm{of}\;\mathrm{covered}\;\mathrm{nodes}\right\}\wedge \\ \mathrm{minimize}\left\{\mathrm{time}\;\mathrm{spent}\;\mathrm{per}\;\mathrm{covered}\;\mathrm{node}\right\}\; \end{array}$$

(6) $$\mathrm{Time}-\mathrm{Coverage}\;\mathrm{Efficiency}\approx \frac{\mathrm{the}\;\mathrm{number}\;\mathrm{of}\;\mathrm{covered}\;{\mathrm{nodes}}^{2}}{\mathrm{time}\;\mathrm{spent}\;\mathrm{per}\;\mathrm{covered}\;{\mathrm{node}}^{\frac{1}{2}}}$$

In (6), we square the number of covered nodes in the numerator of the fraction, because it must be promoted to higher values as much as possible; we take the square root of the time spent per covered node in the denominator because the time spent per covered node has to be minimized as much as possible. In the evaluation and comparison, scenarios which perform better in terms of these metrics—more number of covered nodes and less time spent per covered node—have to be identified and be recommended for use in the operation area. Therefore, we defined the metric as given in (6).

**Figure 7:**
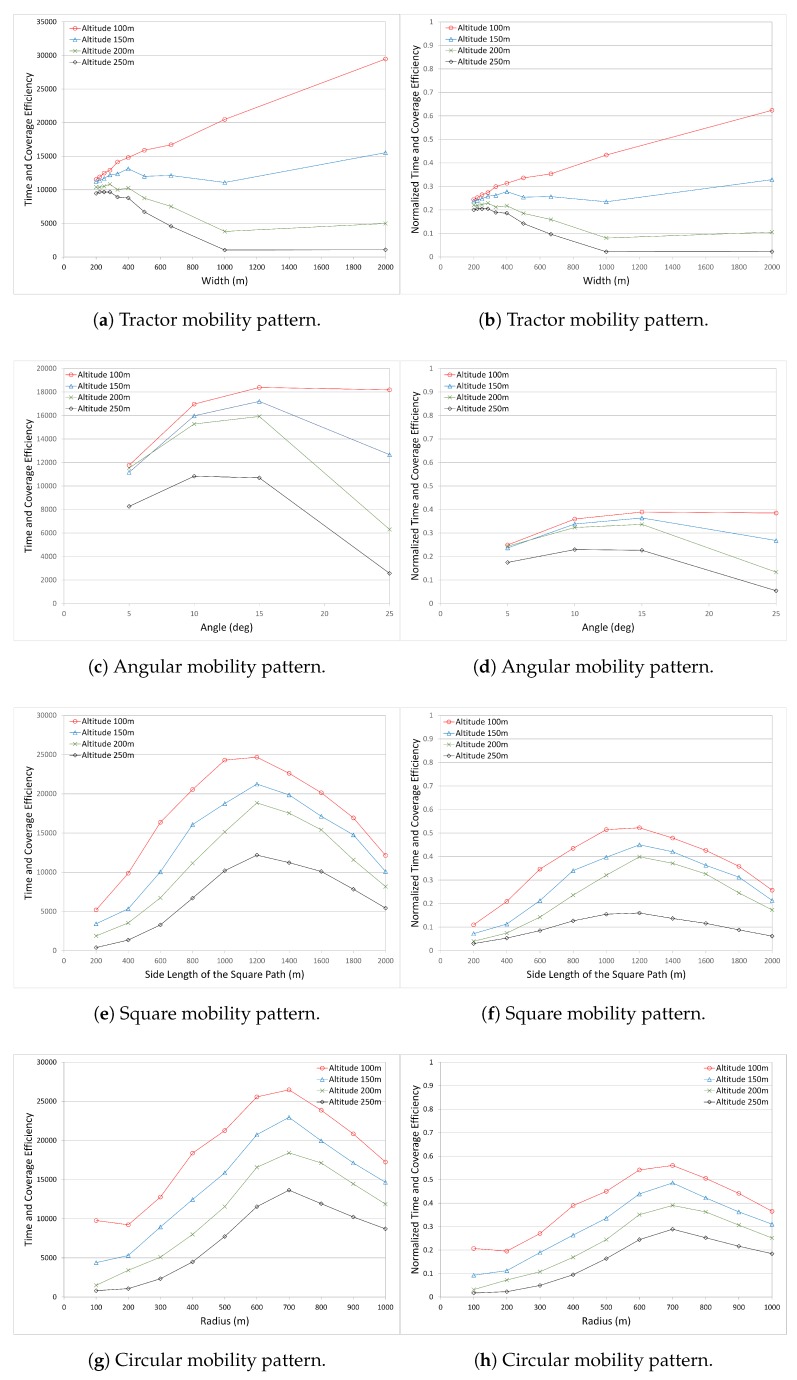
Comparison of Time versus Coverage Efficiency for all mobility patterns.

Figure 7 shows the Time and Coverage Efficiency in all mobility patterns (in the left column). In the Angular mobility pattern, it is seen that although more nodes are covered with the angle value 5${}^{\circ }$ at all altitudes, it is not the most efficient one because of how long it takes to sweep the operation area. With angle 5${}^{\circ }$, more legs are constructed, which increases the total length of the UAV path. This leads to the coverage of more nodes, but longer duration. However, as the angle increases, the number of legs decreases, which eventually decreases the path length of the UAV. It thus takes less time to sweep the area. On the other hand, when the number of covered nodes is considered, there is a slight decrease. It means that with a tradeoff allowing a few nodes to be uncovered, the UAV saves time in collecting data. This is true for angles 10${}^{\circ }$ and 15${}^{\circ }$. For angle 25${}^{\circ }$, although the UAV has the minimum flight path, there is a sharp decrease in the number of covered nodes, especially for altitude 250 m. When the results of the Angular mobility pattern are considered, angle 15${}^{\circ }$ presents the optimum results for time–coverage efficiency. It is also seen that the performance at altitude 250 m is the worst among the others, and for the altitude 250 m, the angle 10${}^{\circ }$ presents better results than the angle 15${}^{\circ }$.

In the Circular mobility pattern, it is clearly seen that the Circular mobility pattern with the radius 700 m is better than other radius values at all altitudes. It can also be verified by looking at two other metrics—the number of covered nodes and the time spent per node graphs.

In the Square mobility pattern (very similar to the circular mobility pattern), it is clearly seen that the square mobility pattern with the side length 1200 m is better than other side length values at all altitudes. This information can also be verified by looking at two other metrics—the number of covered nodes and the time spent per node graphs.

The performance of the Tractor mobility pattern is very different compared to the other mobility patterns. For the 100 m altitude, as the width between the parallel tracks gets larger, time–coverage efficiency increases. The reason is related to the reduced flight time of the UAV by decreasing the path while preserving the number of covered nodes. However, the situation is very different at other altitudes. As the width increases, the time–coverage efficiency value follows a stable pattern for the 150 m altitude and decreases sharply for 200 m and 250 m altitudes. Performance at 250 m altitude is very poor compared to the others.

In all mobility patterns, the UAV altitude 100 m performs better than other altitude values. The reasons are explained previously, and are illustrated in Figure 1b and Figure 2. However, for a specific mobility pattern and depending on the altitude, the most appropriate performance parameter that presents the highest results may differ. For example, in the Angular mobility pattern, the most appropriate angle value for the best performance is 10${}^{\circ }$ for the altitude 250 m, whereas the most appropriate angle value for the best performance is 15${}^{\circ }$ for the altitude 200 m. Depending on the operation altitude of the UAV and the mobility pattern, the UAV operator can choose the right parameters to get better performance results.

Although the time–coverage efficiency metric provides many valuable information, it is necessary to normalize the results to allow them to be compared with each other. Equation (7) represents the efficiency of normalized time and the efficiency of normalized coverage of each mobility pattern.

(7) $$\begin{array}{c} \mathrm{Normalized}\;\mathrm{Time}-\mathrm{Coverage}\;\mathrm{Efficiency}\approx \left[\frac{{\left(\frac{\mathrm{number}\;\mathrm{of}\;\mathrm{covered}\;\mathrm{nodes}\;}{\max_{\mathrm{all}\;\mathrm{scenarios}}\mathrm{number}\;\mathrm{of}\;\mathrm{covered}\;\mathrm{nodes}}\right)}^{2}}{{\left(\frac{\mathrm{time}\;\mathrm{spent}\;\mathrm{per}\;\mathrm{covered}\;\mathrm{node}\;}{\min_{\mathrm{all}\;\mathrm{scenarios}}\mathrm{time}\;\mathrm{spent}\;\mathrm{per}\;\mathrm{covered}\;\mathrm{node}}\right)}^{\frac{1}{2}}}\right] \end{array}$$

Equation (7) is similar to (6), where numerator and the denominator are divided by new values. The numerator is divided by the maximum number of covered nodes among all scenarios, including all mobility patterns and altitudes to normalize this value between 0 and 1. Similarly, the denominator “time spent per covered node” is divided by the minimum time spent per covered node among all scenarios, including all mobility patterns and altitudes to normalize this value. Overall results will be scaled varying between 0 and 1 to let all results be compared in the same scale.

Figure 7 shows the Normalized Time and Coverage Efficiency in all mobility patterns (in the right column). It reveals the details and the hidden points in the comparison. For example, although the results of the Circular and Square mobility patterns are similar to each other where the curves present similar patterns, there are differences in the performance. For these two mobility patterns, the curve pattern similarity is seen in the number of covered nodes graphs. However, we are not able to see the difference and its magnitude in those graphs. With the use of Normalized Time–Coverage Efficiency presented in Figure 7, we are able to see the differences and their magnitudes. Hence, more accurate decisions can be made by decision makers (e.g., UAV operators).

When the graphs are compared with each other, it is seen that, generally, the Circle and Square mobility patterns are superior to the others, where the Circle mobility pattern is the most superior. The Tractor mobility pattern performs better only at the altitude 100 m. At other altitudes, it does not perform well compared to the others, due to the increases in path length and operation duration in the Tractor mobility pattern.

On the other hand, the graphs in Figure 7 are very useful to compare the performance of the mobility patterns at each altitude. For example, the question could be “which mobility patterns perform better and at what parameter values for the UAV altitude value 100 m?” The answer will be the tractor mobility with width value 2000 m. However, the UAVs may not fly at this altitude at every area or zone. For the same question, if the altitude is increased to 150 m, then the circle mobility has to be selected with the radius of 700 m. If the altitude is increased to 200 m, either the square mobility with the side length 1200 m or the circular mobility with the radius 700 m can be selected.

If only two mobility patterns are considered (e.g., Angular and Tractor mobility patterns), for some altitudes, the Angular mobility pattern performs better and for the other altitudes the Tractor mobility pattern performs better.

As can be seen, each mobility pattern presents different performance results. In making the decision to select the best and most appropriate mobility pattern for application in the operation area, there could be two main objectives: first, covering the maximum nodes; second, spending less time in the operation area with maximum utilization. By defining the Time–Coverage Efficiency metric, we address this problem by finding the most appropriate mobility pattern with the best performance results.

## 5. Conclusions

In large area and dense deployment WSNs, gathering data from each sensor node becomes an important issue. Providing connectivity for access to each sensor node and gathering data in a short time are challenging and contradicting tasks. We consider these two joint tasks as two joint research questions. In this paper, we aimed to find optimal solutions for these research questions; namely, both accessing each sensor node and collecting data within time limits.

For the first research question (i.e., in a large-scale operation area), using UAV as mobile sink is one of the best and most efficient ways to provide better coverage and connectivity. With the use of mobility patterns, the operation area can be covered methodically. However, the coverage depends on some fixed operational parameters of the UAV, such as speed and altitude. If a well-defined clustering algorithm is used in the WSN, then we can choose the best-performing method among well-known mobility patterns, or may construct a new UAV path according to our needs. In this research, the mobility patterns and their effects on the performance have been explored. It is seen that depending on the mobility pattern, the number of clusters and the number of accessed sensor nodes varies. One important factor that affects the performance is the applied clustering algorithm in WSN. We have used a well-known and well-balancing clustering algorithm in our experiments. It is seen that the clustering algorithm significantly improves the performance. The major inference obtained from the experiments is that the mobility pattern has to be selected considering the clustering algorithm in WSN, or vice versa; based on the mobility pattern of the UAV, an appropriate clustering algorithm can be implemented at the WSN.

The second research question is related to the operation time. The operation time of the UAV can be a cost factor, or the UAV might be incapable of completing the mission due to the time limitations (i.e., endurance). Considering the joint research question, the solution is to cover as many nodes as possible within a limited period of time. Therefore, a trade-off arises as a consequence between the operation time of the UAV and the covered (accessed) nodes. The best appropriate approach can be selected considering the main objective of the WSN and the operational characteristics and maneuvers of the UAV. In this study, we address this problem and define a new metric for time efficiency versus coverage efficiency. We have shown that although one particular UAV mobility pattern (i.e., Tractor mobility pattern) is more efficient for WSN coverage, another pattern (i.e., Circular mobility pattern) is much more efficient for time–coverage efficiency. It is notable that the clustering algorithm applied in WSN plays a key role for performance efficiency.

The performance of the UAV integrated WSN and applied mobility patterns are evaluated with various scenarios with a realistic simulation environment. The simulation environment is constructed with OMNET++ and MiXiM framework with realistic MAC and physical layers and real path loss values. This study contributes to enhance the coverage and connectivity in large-scale WSNs with the use of the UAV. It is one of the first studies that considers the effect of different UAV mobility patterns in conjunction with cluster formation.

Considering the studies in the literature, we extended the use of UAV in WSN architecture to a further level. In addition to analyzing the effects of UAV mobility patterns on data collection in WSN, we have introduced another aspect to the selection of the appropriate mobility pattern to obtain expected performance. As is indicated many times, the clustering and the CH election algorithms which consider the UAV mobility pattern play an important role in the enhancement of the performance results. More efficient clustering and CH election algorithms can be proposed to get better performance results. In this research, we have implemented well-known mobility patterns in the evaluations. Optimal waypoint planning and constructing the optimal clusters based on that UAV path remain as open research studies.

## Figures and Tables

**Table 1:** Simulation parameters.

Parameter	Value
Sink Velocity	20 m/s
Sink Altitude	100 m, 150 m, 200 m, 250 m
Sink Beacon Period	2 s
Number of Nodes	200
Max. Transmission Range	250 m
Simulation Area	2000 m × 2000 m

## References

[1] Akyildiz I.F., Su W., Sankarasubramaniam Y., Cayirci E. (2002). Wireless Sensor Networks: A Survey. Comput. Netw..

[2] Yoneki E., Bacon J. (2005). A Survey of Wireless Sensor Network Technologies: Research Trends and Middleware’s Role.

[3] Abbasi A.A., Younis M. (2007). A survey on clustering algorithms for wireless sensor networks. Comput. Commun..

[4] Luo C., Wu F., Sun J., Chen C.W. Compressive Data Gathering for Large-scale Wireless Sensor Networks. Proceedings of the 15th Annual International Conference on Mobile Computing and Networking.

[5] Das A., Dutta D. (2005). Data Acquisition in Multiple-sink Sensor Networks. SIGMOBILE Mob. Comput. Commun. Rev..

[6] Akkaya K., Younis M., Bangad M. (2005). Sink repositioning for enhanced performance in wireless sensor networks. Comput. Netw..

[7] Somasundara A.A., Ramamoorthy A., Srivastava M.B. Mobile element scheduling for efficient data collection in wireless sensor networks with dynamic deadlines. Proceedings of the 25th IEEE International Real-Time Systems Symposium.

[8] Turgut D., Bölöni L. (2009). Heuristic Approaches for Transmission Scheduling in Sensor Networks with Multiple Mobile Sinks. Comput. J..

[9] Elfes A., Bueno S.S., Bergerman M., Ramos J.G. A semi-autonomous robotic airship for environmental monitoring missions. Proceedings of the 1998 IEEE International Conference on Robotics and Automation.

[10] Dang P., Lewis F.L., Popa D.O. Dynamic Localization of Air-Ground Wireless Sensor Networks. Proceedings of the 2006 14th Mediterranean Conference on Control and Automation.

[11] Erman A.T., van Hoesel L., Havinga P., Wu J. (2008). Enabling mobility in heterogeneous wireless sensor networks cooperating with UAVs for mission-critical management. IEEE Wirel. Commun..

[12] Okcu H., Soyturk M. (2014). Distributed clustering approach for UAV integrated wireless sensor networks. Int. J. Ad Hoc Ubiquitous Comput..

[13] Cobano J.A., Martínez-de Dios J.R., Conde R., Sánchez-Matamoros J.M., Ollero A. (2010). Data Retrieving From Heterogeneous Wireless Sensor Network Nodes Using UAVs. J. Intell. Robot. Syst..

[14] Martinez-de Dios J.R., Lferd K., de San Bernabé A., Núñez G., Torres-González A., Ollero A. (2013). Cooperation Between UAS and Wireless Sensor Networks for Efficient Data Collection in Large Environments. J. Intell. Robot. Syst..

[15] Marta M., Cardei M. Using sink mobility to increase wireless sensor networks lifetime. Proceedings of the 2008 International Symposium on a World of Wireless, Mobile and Multimedia Networks.

[16] Restuccia F., Das S.K. Lifetime optimization with QoS of sensor networks with uncontrollable mobile sinks. Proceedings of the 2015 IEEE 16th International Symposium on A World of Wireless, Mobile and Multimedia Networks (WoWMoM).

[17] Heinzelman W.B., Chandrakasan A.P., Balakrishnan H. (2002). An application-specific protocol architecture for wireless microsensor networks. IEEE Trans. Wirel. Commun..

[18] Younis O., Fahmy S. (2004). HEED: A Hybrid, Energy-Efficient, Distributed Clustering Approach for Ad Hoc Sensor Networks. IEEE Trans. Mob. Comput..

[19] Xu J., Solmaz G., Rahmatizadeh R., Turgut D., Bölöni L. Animal monitoring with unmanned aerial vehicle-aided wireless sensor networks. Proceedings of the 2015 IEEE 40th Conference on Local Computer Networks (LCN).

[20] Sayyed A., de Araújo G.M., Bodanese J.P., Becker L.B. (2015). Dual-Stack Single-Radio Communication Architecture for UAV Acting As a Mobile Node to Collect Data in WSNs. Sensors.

[21] Heinzelman W.R., Chandrakasan A., Balakrishnan H. Energy-Efficient Communication Protocol for Wireless Microsensor Networks. Proceedings of the 33rd Hawaii International Conference on System Sciences.

[22] Younis O., Fahmy S. Distributed clustering in ad-hoc sensor networks: A hybrid, energy-efficient approach. Proceedings of the INFOCOM 2004.

[23] Huang H., Wu J. A probabilistic clustering algorithm in wireless sensor networks. Proceedings of the 2005 IEEE 62nd Vehicular Technology Conference (VTC-2005-Fall).

[24] Li H., Wang L., Pang S., Towhidnejad M. A Cross-Layer Design for Data Collecting of the UAV-Wireless Sensor Network System. Proceedings of the 2014 12th IEEE International Conference on Embedded and Ubiquitous Computing.

[25] Mori S. Cooperative sensing data collecting framework by using unmanned aircraft vehicle in wireless sensor network. Proceedings of the 2016 IEEE International Conference on Communications (ICC).

[26] Wang C., Ma F., Yan J., De D., Das S.K. (2015). Efficient Aerial Data Collection with UAV in Large-Scale Wireless Sensor Networks. Int. J. Distrib. Sens. Netw..

[27] Ho D.T., Grøtli E.I., Sujit P.B., Johansen T.A., Sousa J.B. (2015). Optimization of Wireless Sensor Network and UAV Data Acquisition. J. Intell. Robot. Syst..

[28] Itkin M., Kim M., Park Y. (2016). Development of Cloud-Based UAV Monitoring and Management System. Sensors.

[29] Wei P., Gu Q., Sun D. Wireless sensor network data collection by connected cooperative UAVs. Proceedings of the 2013 American Control Conference.

[30] Costa F.G., Ueyama J., Braun T., Pessin G., Osório F.S., Vargas P.A. The use of unmanned aerial vehicles and wireless sensor network in agricultural applications. Proceedings of the 2012 IEEE International Geoscience and Remote Sensing Symposium.

[31] Polo J., Hornero G., Duijneveld C., García A., Casas O. (2015). Design of a low-cost Wireless Sensor Network with {UAV} mobile node for agricultural applications. Comput. Electron. Agric..

[32] Tuna G., Mumcu T.V., Gulez K., Gungor V.C., Erturk H., Huang D.S., Gupta P., Zhang X., Premaratne P. (2012). Unmanned Aerial Vehicle-Aided Wireless Sensor Network Deployment System for Post-disaster Monitoring. Emerging Intelligent Computing Technology and Applications: 8th International Conference, ICIC 2012, Huangshan, China, 25–29 July 2012. Proceedings.

[33] Erdelj M., Natalizio E. UAV-assisted disaster management: Applications and open issues. Proceedings of the 2016 International Conference on Computing, Networking and Communications (ICNC).

[34] Dong M., Ota K., Lin M., Tang Z., Du S., Zhu H. (2014). UAV-assisted data gathering in wireless sensor networks. J Supercomput..

[35] Jawhar I., Mohamed N., Al-Jaroodi J. UAV-based data communication in wireless sensor networks: Models and strategies. Proceedings of the 2015 International Conference on Unmanned Aircraft Systems (ICUAS).

[36] OMNeT++ Discrete Event Simulator. https://omnetpp.org/.

[37] MiXiM Simulation Framework for Wireless and Mobile Networks. http://mixim.sourceforge.net/.

[38] Köpke A., Swigulski M., Wessel K., Willkomm D., Haneveld P.T.K., Parker T.E.V., Visser O.W., Lichte H.S., Valentin S. Simulating Wireless and Mobile Networks in OMNeT++ the MiXiM Vision. Proceedings of the 1st International Conference on Simulation Tools and Techniques for Communications, Networks and Systems & Workshops.

[39] TI CC2420 2.4 GHz IEEE 802.15.4 / ZigBee-Ready RF Transceiver. http://www.ti.com/lit/ds/symlink/cc2420.pdf.

[40] Bayraktar Mini-UAV Datasheet. http://baykarmakina.com/wp-content/uploads/2015/01/minip.pdf.

